# mtDNA haplogroup A enhances the effect of obesity on the risk of knee OA in a Mexican population

**DOI:** 10.1038/s41598-022-09265-y

**Published:** 2022-03-25

**Authors:** Paula Ramos-Louro, Rubén Daniel Arellano Pérez Vertti, Alberto López Reyes, Gabriela Angélica Martínez-Nava, Rolando Espinosa, Carlos Pineda, Faviel Francisco González Galarza, Rafael Argüello Astorga, Lizette Sarai Aguilar Muñiz, Fernando Hernández Terán, Nancy Marbella Parra Torres, Alejandro Durán Sotuela, Mercedes Fernández-Moreno, Vanesa Balboa Barreiro, Francisco J. Blanco, Ignacio Rego-Pérez

**Affiliations:** 1 Grupo de Investigación en Reumatología (GIR), Instituto de Investigación Biomédica de A Coruña (INIBIC), Complexo Hospitalario Universitario de A Coruña (CHUAC), Sergas, Universidade da Coruña (UDC), C/ As Xubias de Arriba 84, 15006 A Coruña, Spain; 2 Facultad de Medicina, Universidad Autónoma de Coahuila, Torreon Coahuila, Mexico; 3 Laboratorio de Gerociencias, Departamento de Reumatología Dirección General, Instituto Nacional de Rehabilitación Luis Guillermo Ibarra Ibarra, Secretaría de Salud, Mexico, Mexico; 4 División de Ciencias de La Salud, Universidad Intercultural del Estado de Puebla, Huehuetla, Mexico; 5 Grupo de Investigación en Reumatología y Salud, Departamento de Fisioterapia, Medicina y Ciencias Biomédicas, Facultad de Fisioterapia, Universidade da Coruña (UDC), Campus de Oza, 15008 A Coruña, Spain

**Keywords:** Genetics, Genetic markers, Sequencing

## Abstract

To evaluate the influence of mitochondrial DNA haplogroups on the risk of knee OA in terms of their interaction with obesity, in a population from Mexico. Samples were obtained from (n = 353) knee OA patients (KL grade ≥ I) and (n = 364) healthy controls (KL grade = 0) from Mexico city and Torreon (Mexico). Both Caucasian and Amerindian mtDNA haplogroups were assigned by single base extension assay. A set of clinical and demographic variables, including obesity status, were considered to perform appropriate statistical approaches, including chi-square contingency tables, regression models and interaction analyses. To ensure the robustness of the predictive model, a statistical cross-validation strategy of B = 1000 iterations was used. All the analyses were performed using boot, GmAMisc and epiR package from R software v4.0.2 and SPSS software v24. The frequency distribution of the mtDNA haplogroups between OA patients and healthy controls for obese and non-obese groups showed the haplogroup A as significantly over-represented in knee OA patients within the obese group (OR 2.23; 95% CI 1.22–4.05; p-value = 0.008). The subsequent logistic regression analysis, including as covariate the interaction between obesity and mtDNA haplogroup A, supported the significant association of this interaction (OR 2.57; 95% CI 1.24–5.32; p-value = 0.011). The statistical cross-validation strategy confirmed the robustness of the regression model. The data presented here indicate a link between obesity in knee OA patients and mtDNA haplogroup A.

## Introduction

Osteoarthritis (OA) is a chronic musculoskeletal disease with a polygenic and heterogeneous nature that involves movable joints. The disease is characterized by cell stress and extracellular matrix degradation in which pro-inflammatory pathways of innate immunity are activated. After an initial molecular derangement consisted in an abnormal tissue metabolism, different physiologic and anatomic derangements take place, including cartilage degradation, bone remodeling, osteophyte formation, joint inflammation and loss of normal function^1^. All these features lead to consider OA as a severe disease of the whole joint as an organ^2^, with still large unmet clinical needs.

It is well known that age is one of the most evident risk factors for OA, presumably as a result of the cumulative exposure, not only to age-related changes in joint structures, but also to different risk factors^3^. Specifically, for knee OA, a variety of moderate to strong risk factors include female gender, knee malalignment, previous knee injury and obesity^4,5^. Precisely, OA, as well as diabetes, were responsible for the largest increase in disability during the period 2005–2015. Among the causes of this increase, both the global increase in life expectancy and obesity pandemic stand out^6^.

The impact of obesity on the risk of OA is not only related to the increase in mechanical forces on weight-bearing joints, but also to a systemic link between obesity and OA. Proof of the latter is the association between obesity and hand OA^7^. On the one hand, excess weight results in cartilage compression that is detected by mechanoreceptors on chondrocyte surfaces, leading to the activation of inflammatory signaling cascades^8^; on the other hand, the systemic link between obesity and OA seems to be mediated, at least in part, by adipokines^9^. Adipokines include a variety of pro-inflammatory factors that contribute to the low-grade inflammatory state in obese individuals^10^. In addition, adipokines released by osteoarthritic chondrocytes induce inflammation and contribute to the formation of osteophytes^11^.

Mitochondrial dysfunction is implicated in the development of OA and obesity. Regarding OA, mitochondrial dysfunction is well documented, and causes an increase of pro-inflammatory cytokines and metalloproteinases as well as an excessive chondrocyte apoptosis and enhanced reactive oxygen species (ROS) production, ultimately leading to a decreased ATP production, mtDNA damage and cartilage degeneration^12,13^. In the case of obesity, mitochondrial dysfunction can potentially affect glycolysis^14^ and, on the other hand, the metabolic oversupply, or overabundance of energetic substrates such as lipids or glucose, leads to mitochondrial overheating. This state is potentially cytotoxic, leading to oxidative damage, mitochondrial fission and fragmentation, altering mtDNA and compromising cellular function^15^.

In terms of mtDNA variation, different independent studies showed some associations between mtDNA haplogroups and both OA and obesity. Specifically, subjects with mtDNA haplogroups belonging to the Caucasian mtDNA cluster JT have lower risk of developing incidence knee OA and progression, when compared with subjects with haplogroup H/HV^16,17^. Moreover, subjects with Asian haplogroup B were at higher risk for new development of knee OA^18^, and southern Chinese patients with haplogroup G exhibited a higher occurrence of knee OA as well as higher severity progression of knee OA^19^. On the other hand, mtDNA variants T and J have been linked to opposite obesity-related effects. mtDNA haplogroup T have been associated with obesity in Austrian and Italian populations^20,21^ and mtDNA haplogroup J was negatively associated with obesity in Italians^20^. In addition, haplogroups belonging to the mtDNA cluster IWX have also been associated with an increased risk of incident obesity at 8 years in patients from the osteoarthritis initiative (OAI) cohort^14^ but, contrarily, haplogroup X was associated with lower BMI and body fat mass in US subjects of northern European ancestry^22^. Hidden or unknown potential genetic mitochondrial-nucleus interactions and/or environmental variables could be behind this complex interplay between mitochondrial function and obesity.

In the work presented here, we aimed to analyze the influence of mtDNA haplogroups on the risk of knee OA in two populations from Mexico, specifically in terms of the interaction between these mtDNA variants and obesity.

## Methods

### Patients and controls

A total of 353 unrelated patients recruited from the Rheumatology service of National Rehabilitation Institute of Mexico DF and the Faculty of Medicine of Torreon (Autonomous University of Coahuila), and diagnosed as having primary knee OA, were included in the present study. Those patients meeting the inclusion criteria included individuals of both sexes (260 females; 93 males), with a mean age of 58.5 ± 15.6 years, and diagnosed with knee OA following the American College of Rheumatology (ACR) criteria. The 364 donors from the same locations who met the inclusion criteria for healthy subjects included individuals of both sexes (242 females; 122 males), with a mean age of 50.0 ± 12.7 years, and lacked the ACR criteria for knee OA. Knee radiographs from the entire cohort, acquired in anteroposterior and lateral projection in flexion at 30 degrees, were classified according to Kellgren and Lawrence scoring from Grade 0 to Grade IV. Subjects diagnosed of rheumatoid arthritis, autoimmune disease-associated arthritis, infectious arthritis and post-traumatic dysplasia, congenital or skeletal, were excluded. This study was conducted following the good clinical practice and the Declaration of Helsinki. An informed consent was obtained from each patient before entering the study. The ethics and research committees of the National Institute of Rehabilitation (INR: 08/07) and the Faculty of Medicine of Torreon from the Autonomous University of Coahuila (C.B/04-10-17) approved the study protocol.

### mtDNA haplogroups genotyping

In this study, we used the single base extension (SBE) assay and conventional sequencing techniques to assess the most common European and Amerindian mtDNA haplogroups. The polymorphic sites analyzed to assign the Amerindian haplogroups are described in Supplementary Table 1.

The haplogroup genotyping strategy consisted, firstly, in the assignment of Caucasian mtDNA haplogroups following the protocol described elsewhere^23^. Then, the assignment of Amerindian haplogroups was performed in those subjects that did not carry Caucasian haplogroups. For this, six specific primers were designed to amplify the mtDNA fragments that contain each of the informative polymorphisms that characterize four of the five major Amerindian mtDNA haplogroups (A, C, D and X) in one multiplex reaction. In addition, another six specific SBE primers were also designed to identify each of the diagnostic SNPs. The polymorphic site characteristic of the haplogroup B consists in a 9 bp-deletion, therefore the fragment containing this polymorphism was amplified separately and further identified by conventional capillary sequencing using BigDye v3.1 chemistry^®^ (Applied Biosystems). The sequences for PCR and SBE primers are listed in Supplementary Table 2.

The multiplex PCR mixture consisted of a final concentration of 1× reaction buffer (Bioline, London, UK), 0.2 mM of each deoxynucleotide (dNTP) (Bioline), 1.5 mM MgCl_2_ (Bioline), 0.025 U/µL of BioTaq DNA polymerase (Bioline) and 0.3 µM of each primer in a volume of 50 µL. Genomic DNA (75 ng), isolated from peripheral blood using a commercial kit (QIAamp DNA blood Mini Kit, QIAGEN, Germany), was added to the mixture and amplified as follows: 94 °C for 5 min, 35 cycles at 95 °C for 60 s, 50 °C for 60 s and 72 °C for 60 s, and a final extension at 70 °C for 10 min^23^. To remove primers and unincorporated dNTPs, multiplex PCR products were treated with ExoSap-IT (Amersham; London, UK) following the manufacturer’s recommendations.

Multiplex SBE reaction was carried out in a final volume of 10 µL, by adding 1.5 µL of SNaPshot^®^ Multiplex Kit (Applied Biosystems), 2.1 µL of purified PCR product and a final concentration of 0.2 µM of the SBE primers mixture. To reach the final volume, distilled, deionized water (ddH_2_O) was added. The thermal cycling conditions for SBE were as follows: 96 °C for 60 s and 25 cycles at 96 °C for 10 s, 60 °C for 5 s and 60 °C for 30 s. To remove unincorporated dideoxynucleotides (ddNTPs), the SBE reaction products were treated with a thermosensitive alkaline phosphatase (FastAP) (ThermoFisher Scientific) following the manufacturer’s instructions. Finally, 9.5 µL of Hi-Di™ Formamide (Applied Biosystems), 0.15 µL of size internal standard (120 Liz Size Standard from Applied Biosystems) and 0.35 µL of purified SBE product were mixed and denatured at 95 °C for five minutes prior to loading into an ABI 3130xl genetic analyzer (Applied Biosystems). Once the runs were finished, the data were analyzed using GeneMapper v3.5 software (Applied Biosystems), which assigns the different alleles (SNPs) in each *locus* using a reference sequence that encompasses all the allelic variants for each *locus*^23^.

### Statistical analyses

Statistical analyses were performed using SPSS software v24 and epiR package from R software v4.0.2. Statistical significance was declared at p < 0.05. First, a descriptive analysis of the study population grouped by Amerindian mtDNA haplogroups was performed for all the clinical and demographic variables. Then, a univariate analysis of risk factors affecting knee OA was calculated, followed by a logistic regression model including the mtDNA haplogroups as covariate, and considering the most common Amerindian haplogroup A as the reference haplogroup. Those minor subjects harbouring Caucasian haplogroups were classified in the group “Others”. To analyze the interactions between Amerindian mtDNA haplogroups and obesity, the cohort was splitted into obese (BMI ≥ 30 kg/m^2^) and non-obese (BMI < 30 kg/m^2^) subjects, and the frequency distribution of the mtDNA haplogroups between OA patients and healthy subjects for each scenario (obese and non-obese) was calculated. Finally, the additive interaction between obesity and haplogroups was evaluated by logistic regression. To quantify the additive interaction, three measures were used: relative excess risk due to interaction (RERI), attributable proportion (AP) and Rothman’s synergy index (S), all of them calculated with their 95% confidence intervals using the delta method of Hosmer and Lemeshow.

To explore more in detail this association, two additional interaction analyses were performed: (i) between obesity, KL grade (from KL 0 to KL IV) and haplogroup A; and (ii) between OA diagnosis, BMI categories (normal weight: < 24.9; overweight: 25–29.9; obesity type I: 30–34.9; obesity type II: 35–39.9; obesity type III ≥ 40) and mtDNA haplogroup A. One-factor ANOVA test was used to compare the estimated probabilities.

Finally, an internal validation of the logistic regression model proposed in the study was carried out using the cross-validation procedure. The full dataset was splitted into two random parts, a training (75%) and a validation (25%) portion. The model was fitted on the training set and its performance was evaluated on both the training and the validation portions, using area under the curve (AUC) as performance measure. The model’s estimated coefficients and p-values were stored. This procedure was repeated B = 1000 times, getting a fitting and validation distribution of the AUC values, as well as a fitting distribution of the coefficients and associated p-values. The cross-validation was carried out with the R packages boot and GmAMisc.

## Results

The initial descriptive analysis of the 717 subjects showed a differentially significant association of age with mtDNA haplogroups, and bordering on the statistical significance in the case of gender. For all other variables, including obesity, KL grade and severity (KL 0–II vs KL III–IV), we did not detect significant differences in their distribution among the Amerindian mtDNA haplogroups. Among the Amerindian mtDNA haplogroups, haplogroup A was the most frequent within Mexican population, reaching a frequency of 42% in the general population (Table 1).

**Table 1:** Demographic characteristics of the study population grouped by American mitochondrial DNA (mtDNA) haplogroups.

mtDNA haplogroups							
Characteristic	A (N = 301, 42.0%)	B (N = 156, 21.8%)	C (N = 113, 15.8%)	D (N = 51, 7.1%)	Others (N = 96, 13.4%)	p-value	Total (N = 717)
Age at baseline (years)	52.3 ± 14.2	54.3 ± 15.4	56.3 ± 15.8	52.7 ± 14.7	58.2 ± 13.6	0.005*	54.2 ± 14.8
**Gender**						0.052^#^	
Male	90 (29.9)	55 (35.3)	29 (25.7)	8 (15.7)	33 (34.4)		215 (30.0)
Female	211 (70.1)	101 (64.7)	84 (74.3)	43 (84.3)	63 (65.6)		502 (70.0)
**Obesity (BMI ≥ 30 kg/m**^**2**^**)**						0.311^#^	
Obese	96 (31.9)	35 (22.4)	30 (26.5)	15 (29.4)	28 (29.2)		204 (28.5)
Non-obese	205 (68.1)	121 (77.6)	83 (73.5)	36 (70.6)	68 (70.8)		513 (71.5)
KL grade (worst knee)**						0.681^#^	
0	148 (49.2)	83 (53.2)	57 (50.4)	27 (52.9)	49 (51.0)		364 (50.8)
I	39 (13.0)	22 (14.1)	16 (14.2)	8 (15.7)	7 (7.3)		92 (12.8)
II	60 (19.9)	20 (12.8)	17 (15.0)	8 (15.7)	18 (18.8)		123 (17.2)
III	40 (13.3)	17 (10.9)	16(14.2)	6 (11.8)	17 (17.7)		96 (13.4)
IV	14 (4.7)	14 (9.0)	7 (6.2)	2 (3.9)	5 (5.2)		42 (5.9)
**Radiographic severity**						0.789^#^	
KL 0–II	247 (82.1)	125 (80.1)	90 (79.6)	43 (84.3)	74 (77.1)		579 (80.8)
KL III–IV	54 (17.9)	31 (19.9)	23 (20.4)	8 (15.7)	22 (22.9)		138 (19.2)

Values are mean ± standard deviation or number of patients with percentage in parentheses.

*BMI* body mass index, *KL* Kellgren and Lawrence.

*Kruskal–Wallis test for comparison between mtDNA haplogorups.

**The worst knee is the knee with the highest KL grade; significant p-values are in bold.

^#^Chi-square test.

The univariate analysis of potential risk factors for knee OA showed that older age (OR 1.04; 95% CI 1.03–1.05; p-value < 0.001), female gender (OR 1.41; 95% CI 1.02–1.94; p-value = 0.036) and obesity (OR 2.57; 95% CI 1.83–3.60; p-value < 0.001) increase the risk of developing knee OA. On the contrary, none of the haplogroups showed any association with the risk of developing knee OA (Table 2). The subsequent logistic regression analysis confirmed the associations of older age (OR 1.05; 95% CI 1.04–1.06; p-value < 0.001), female gender (OR 1.76; 95% CI 1.24–2.51 p-value = 0.002) and obesity (OR 2.87; 95% CI 2.01–4.10; p-value < 0.001), as well as the absence of association of the mtDNA haplogroups after comparing each haplogroup with the most prevalent A haplogroup (Table 3).

**Table 2:** Univariate analysis of risk factors affecting knee osteoarthritis (KL grade ≥ I).

Characteristic	Healthy (N = 364)	OA (N = 353)	p-value	OR	95% CI
Age at baseline (years)	50.0 ± 12.7	58.5 ± 15.6	** < 0.001**	1.04	1.03–1.05
**Gender**					
Male	122 (33.5)	93 (26.3)		1	
Female	242 (66.5)	260 (73.7)	**0.036**	1.41	1.02–1.94
**Obesity (BMI ≥ 30 kg/m**^**2**^**)**					
Obese	70 (19.2)	134 (38.0)	** < 0.001**	2.57	1.83–3.60
No-obese	294 (80.8)	219 (62.0)		1	
mtDNA haplogroups*					
A	148 (40.7)	153 (43.3)	0.467	1.12	0.83–1.50
B	83 (22.8)	73 (20.7)	0.491	0.88	0.62–1.26
C	57 (15.7)	56 (15.9)	0.940	1.02	0.68–1.52
D	27 (7.4)	24 (6.8)	0.747	0.91	0.51–1.61
Others	49 (13.5)	47 (13.3)	0.954	0.99	0.64–1.52

Values are mean ± standard deviation or number of patients with percentage in parentheses.

*BMI* body mass index, *KL* Kellgren and Lawrence.

*Statistical parameters after comparing the presence of each haplogroup versus its absence; significant p-values are in bold.

**Table 3:** Multivariate logistic regression model to predict the risk of knee OA (KL grade ≥ I) in Mexican population.

Variable	B	Adjusted OR	95% CI	p-value
Age	0.048	1.05	1.04–1.06	** < 0.001**
Gender (female)	0.567	1.76	1.24–2.51	**0.002**
Obesity	1.054	2.87	2.01–4.10	** < 0.001**
mtDNA haplogroups*				
B vs A	− 0.156	0.86	0.56–1.30	0.467
C vs A	− 0.229	0.79	0.50–1.28	0.343
D vs A	− 0.251	0.78	0.41–1.47	0.778
Others vs A	− 0.312	0.73	0.44–1.21	0.732

*B* regression coefficient, *OR* odds ratio, *CI* confidence interval.

*Statistical analyses were performed comparing the frequency distribution of each haplogroup with that of the most common haplogroup A (reference haplogroup).

Statistical significance declared at p < 0.05 (in bold).

As a first step in assessing potential interactions between obesity and haplogroups, we splitted the cohort into obese and non-obese subjects attending to their BMI (≥ 30 kg/m^2^ and < 30 kg/m^2^ respectively). The “Obese group” consisted of 204 subjects (70 healthy and 134 OA) and the “Non-obese group” consisted of 513 (294 healthy and 219 OA). Then, the frequency distribution of the mtDNA haplogroups between OA patients and healthy controls for each group was calculated, and the results obtained showed the haplogroup A significantly over-represented in knee OA patients within the “Obese group” (OR 2.23; 95% CI 1.22–4.05; p-value = 0.008) (Table 4). The subsequent logistic regression analysis, including as covariate the interaction between obesity and mtDNA haplogroup A, in addition to age, gender, obesity and mtDNA haplogroup A, confirmed the significant association of this interaction (OR 2.57; 95% CI 1.24–5.32; p-value = 0.011) (Table 5). Moreover, this association was subsequently confirmed after applying the internal validation strategy (Table 5). To quantify the effect of the interaction, we create a dummy variable with 4 categories combining obesity and haplogroup A, and considering “No-obese + no haplogroup A” as the reference category. The resultant interaction parameters confirmed the presence of a positive interaction between obesity and haplogroup A on the risk of knee OA (Supplementary Table 3). Specifically, a RERI of 2.23 (95% CI 0.37–4.09) suggests an excess risk of 2.23 due to interaction. An AP of 0.6 (95% CI 0.34–0.87) denotes that 60% of the OR of the subjects with a diagnosis of knee OA exposed to both factors is attributed to this interaction. An S value of 5.80 (95% CI 0.95–35.35) indicates that the risk of knee OA in obese patients with haplogroup A is 5.80 times higher than the sum of the risks of the subjects exposed to a single risk factor, leading to a synergy between obesity and haplogroup (Supplementary Table 3).

**Table 4:** Frequency distribution of American mtDNA haplogroups between osteoarthritic patients and healthy controls stratified by their obesity status.

Obesity status	mtDNA haplogroup	Healthy (N = 364)	OA (N = 353)	p-value*	OR	95% CI
Obese (BMI ≥ 30 kg/m^2^)	A	24 (34.3)	72 (53.7)	**0.008**	2.23	1.22–4.05
	B	14 (20.0)	21 (15.7)	0.436	0.74	0.35–1.57
	C	13 (18.6)	17 (12.7)	0.260	0.64	0.29–1.40
	D	7 (10.0)	8 (6.0)	0.295	0.57	0.20–1.65
	Others	12 (17.1)	16 (11.9)	0.305	0.65	0.29–1.48
Total		70	134			
No-obese (BMI < 30 kg/m^2^)	A	124 (42.2)	81 (37.0)	0.235	0.80	0.56–1.15
	B	69 (23.5)	52 (23.7)	0.942	1.01	0.67–1.53
	C	44 (15.0)	39 (17.8)	0.387	1.23	0.77–1.97
	D	20 (6.8)	16 (7.3)	0.825	1.08	0.55–2.14
	Others	37 (12.6)	31 (14.2)	0.604	1.14	0.69–1.91
Total		294	219			

Values are number of patients with percentage in parentheses.

*BMI* body mass index, *OR* odds ratio, *CI* confidence interval.

*Statistical significance declared at p ≤ 0.01 (in bold) after Bonferroni correction.

**Table 5:** Multivariate logistic regression model to explore the effect of the interaction between obesity and mtDNA haplogroup A on the risk of knee OA in the full dataset of Mexican subjects and results of the internal validation strategy after 1000 iterations.

Variable	Full dataset				1000 Bootstrap samples*		
	B	Adjusted OR	95% CI	p-value	B	Adjusted OR	p-value
Age	0.048	1.05	1.04–1.06	** < 0.001**	0.048	1.05	** < 0.001**
Gender (female)	0.578	1.78	1.25–2.54	**0.001**	0.580	1.79	**0.006**
Obesity	0.646	1.91	1.20–3.04	**0.007**	0.656	1.93	**0.017**
mtDNA haplogroup A	− 0.044	0.96	0.65–1.40	0.822	− 0.040	0.96	0.721
Obesity × mtDNA haplogroup A	0.944	2.57	1.24–5.32	**0.011**	0.945	2.57	**0.029**

	Estimation	Median	95% CI		Median	95% CI	
AUC	0.721	0.723	0.69–0.76		0.713	0.60–0.82	

*BMI* body mass index, *B* regression coefficient, *OR* odds ratio, *CI* confidence interval, *AUC* area under the curve used as performance measure of internal validation.

Statistical significance declared at p < 0.05 (in bold).

*Internal validation with estimated coefficients across B = 1000 iterations reported in 75% of the full sample.

**Figure 1:**
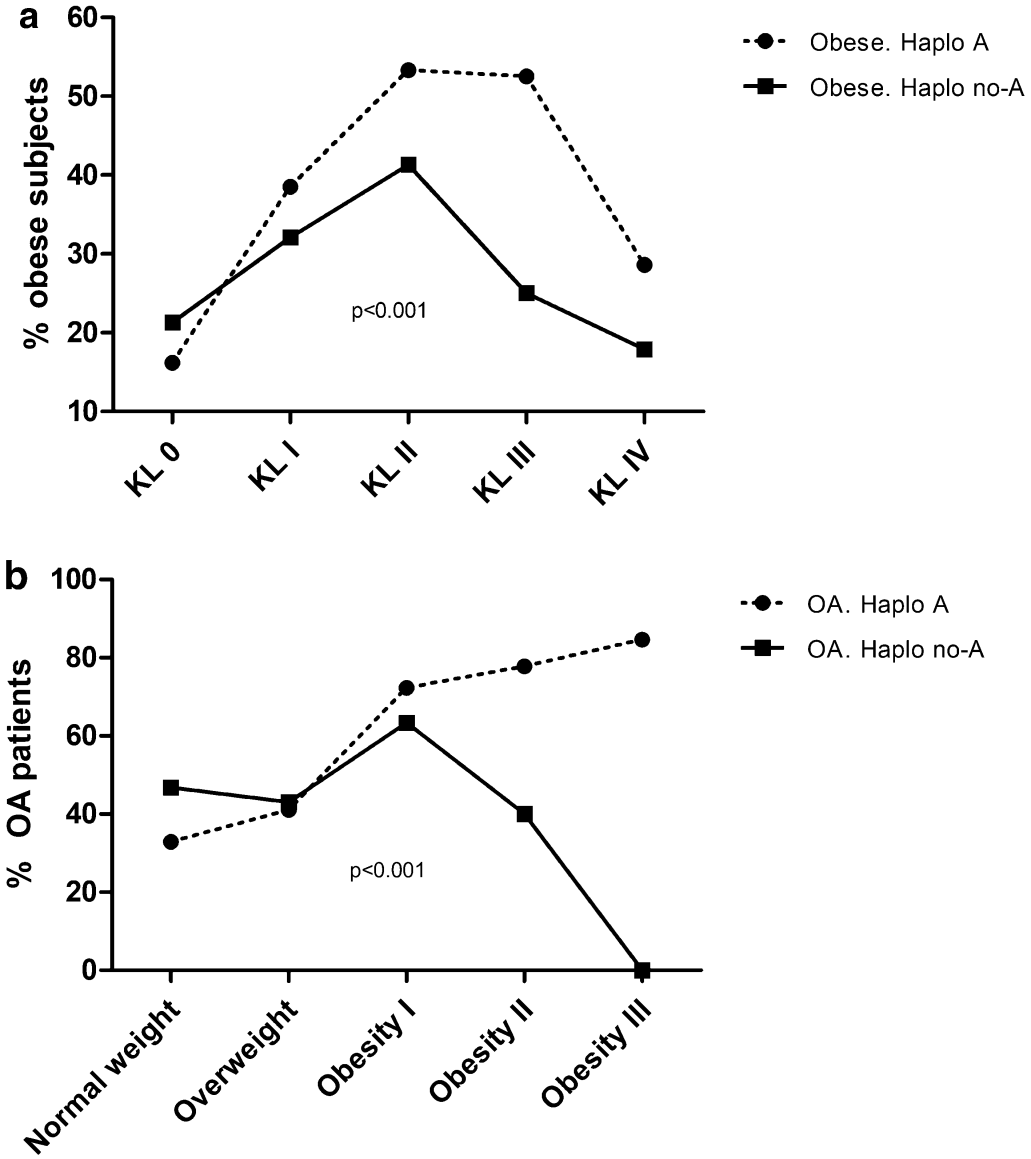
(**a**) Percentage of obese subjects on carriers and non-carriers of mtDNA haplogroup A according to radiological grade from KL 0 to KL IV. One-factor ANOVA test was used to compare the estimated probability of presenting obesity according to KL grade between mtDNA haplogroup A and non-haplogroup A carriers. (**b**) Percentage of OA patients on carriers and non-carriers of mtDNA haplogroup A according to degrees of obesity (normal weight: < 24.9; overweight: 25–29.9; obesity type I: 30–34.9; obesity type II: 35–39.9; obesity type III ≥ 40). One-factor ANOVA test was used to compare the estimated probabilities of presenting OA according to degrees of obesity between mtDNA haplogroup A and non-haplogroup A carriers.

Because of the link observed in OA patients with mtDNA haplogroup A in obese patients, we performed two additional analyses by stratifying subjects according to both their KL grade and degree of obesity. The results of these analyses revealed an increased percentage of obese subjects carrying mtDNA haplogroup A at higher KL grades (p < 0.001) (Fig. 1a), as well as an over-representation of OA patients with mtDNA haplogroup A at higher degrees of obesity (p < 0.001) (Fig. 1b). These results confirm that the estimated probability of presenting OA according to BMI is significantly higher in carriers of mtDNA haplogroup A.

## Discussion

In this work we performed an association study to investigate the impact of specific Amerindian mtDNA haplogroups in the influence that obesity has on the risk of developing knee OA. The results obtained show that obese subjects with haplogroup A have an increased risk of developing knee OA than non-obese subjects with this haplogroup. To our knowledge, this is the first study to analyze the impact of Amerindian mtDNA haplogroups on the risk of obesity-mediated knee OA in Mexican populations.

Although a small percentage (~ 6%) of subjects belong to Caucasian haplogroups, as expected, the haplogroup A was the most prevalent among Mexican subjects (42%). The latter is in agreement with previous studies where different authors analyzed the population structure of Mexican populations based on mtDNA haplogroups. These studies revealed haplogroups A, B, C and D as the most frequent among Mexicans^24,25^. However, it has been postulated that mitochondrial genetic structure in Mexican populations could be influenced by the admixture process that took place after the Spanish conquest in 1519, leading to the development of two groups geographically defined: North-West and Center-South and Southeast^25^. In addition, the frequency of Amerindians haplogroups has been described to be higher in the central region than in Mexico city^24^. Thus, we additionally repeated the analyses after excluding the Mexico city patients and controls, replicating the significant interaction detected when using the entire cohort (Supplementary Tables 4, 5). This result confirms that admixture, if present, does not mask the association detected herein and strengthens the proposal that mtDNA haplogroup A and obesity are key factors leading to knee OA in this population.

Our results reveal an additive interaction between haplogroup A and obesity that enhances the risk of developing knee OA. Through this interaction, not only the percentage of OA patients with mtDNA haplogroup A is significantly higher at higher degrees of obesity, but also the percentage of obese subjects within the haplogroup A is higher among higher KL grades when compared with the rest of haplogroups.

Interactions involving mtDNA haplogroups are not new. Different studies revealed mito-nuclear genetic interactions that modulate the effect of specific nuclear genetic variants on the risk of Alzheimer^26^, Parkinson^27^, multiple sclerosis^28^ or increased BMI in patients with type I Diabetes mellitus^29^. In addition, mitochondrial variation also influences the DNA methylome of articular chondrocytes^30^ as well as global methylation levels in peripheral blood DNA^31^. In the case of this work, potential unexplored synergies between mtDNA haplogroup A and pro-inflammatory factors related to obesity in OA patients, such as adipokines, could be behind this association.

In line with what is stated in the previous paragraph, results obtained in different studies could explain, at least in part, the findings obtained in the present work. This is the case of the interaction between physical activity and haplogroups on serum levels of adiponectin in a Japanese population^32^. Since an increased mitochondrial content is required for adiponectin synthesis, a proposed explanation for this positive correlation is that physical activity is able to increase mitochondrial content in adipocytes^33–36^. The study by Nishida and co-workers revealed a significant interaction between physical activity and mtDNA haplogroups M7a, D and A on serum levels of adiponectin. As a result of this interaction, the positive association of physical activity with adiponectin in subjects carrying haplogroup M7a is attenuated, in comparison to subjects carrying haplogropus D and A^32^.

In addition, associations of Caucasian haplogroups with increased levels of adiponectin have also been described in HIV/hepatitis C virus co-infected patients on highly active antiretroviral therapy. In this case, Caucasian patients from OA-risk mtDNA cluster HV had higher serum levels of adiponectin, whereas patients from OA-protective mtDNA cluster JT had lower serum levels of adiponectin^37^.

Contrarily to most obesity-related pathologies, such as type 2 Diabetes mellitus or atherosclerosis, increased serum levels of adiponectin have been associated with an increased risk of knee OA, although with controversial associations. Specifically, the largest association study to date, including 2402 subjects, revealed a positive association of serum adiponectin levels with radiographic severity of knee OA, in terms of osteophyte and joint space narrow scores, but not with hand OA^38^. Besides, adiponectin levels in OA patients were correlated with pro-inflammatory cytokines in synovial fluid, including Interleukin-1β^39^, as well as with increased degradation markers of aggrecan^40^. Interestingly, adiponectin receptors ADIPOR1 and ADIPOR2 show an increased mRNA and protein expression in chondrocytes obtained from obese patients with OA compared with non-obese OA patients^41^, and vascular cell adhesion protein-1 (VCAM-1) and matrix metalloproteinase-2 (MMP-2) are differentially upregulated by adiponectin in chondrocytes obtained only from obese patients too^41^. Nonetheless, potential synergies between mtDNA haplogroups and any kind of adipokine need to be investigated in detail in this population.

In this work, no direct influence of any of the Amerindian mtDNA haplogroups on the risk of knee OA has been detected, contrarily to other findings in Asian and Caucasian populations^16,42^. However, population-specific associations involving mtDNA haplogroups are not new^43,44^. The existence of potential unknown genetic mitochondrial-nucleus interactions and/or the fact that specific mitochondrial variants that are advantageous in a particular climate zone become neutral or maladaptive in a different environment with new lifestyles, could be behind these controversial associations^45–48^. Specifically, haplogroup A arrived to northern Siberia, together with haplogroups C and D, and colonized the Americas when the Bering land bridge appeared; therefore, these haplogroups would have been subjected to an important cold stress^49,50^. This process led to the development of two very well conserved adaptive mutations found at mtDNAs from haplogroup A: one at the nucleotide position mt4824G (ND2 gene) and the other at mt8794T (ATP6 gene)^50^. Within this scenario, all these features would make haplogroup A an enhancer of the risk of knee OA when combined with obesity in Mexican subjects, but would not show any association by itself with the risk of knee OA.

This work has some limitations that must be drawn. This is a unique study in a discovery cohort of 717 subjects, therefore, although we applied statistical techniques of cross-validation that confirmed the interaction, further replication of these findings in independent larger cohorts of patients would be desirable. On the other hand, we speculate that a synergy between mtDNA haplogroup A and certain pro-inflammatory factors related to obesity, such as adipokines, occurs, however since we did not assess these levels in the serum of the study patients, this aspect deserves further investigation. Finally, the potential population admixture, especially in patients from Mexico city, could mask the results; nevertheless, the replication of these findings after removing from the analyses the subjects from Mexico city demonstrates that admixture, if present, does not mask the results obtained.

In summary, this is the first study to show a significant interaction between Amerindian haplogroups and obesity on the risk of knee OA in populations from Mexico. Specifically, obese subjects with haplogroup A have an enhanced risk of developing knee OA. Confirmation that potential unexplored synergies between mtDNA haplogroup A and pro-inflammatory factors related to obesity are behind this association deserves further investigation.

## Supplementary Information

Supplementary Tables.
