# Supplementary material for: mtDNA haplogroup A enhances the effect of obesity on the risk of knee OA in a Mexican population
Source: Sci Rep. 2022 Mar 25;12:5173. doi: 10.1038/s41598-022-09265-y (PMC8956628; doi:10.1038/s41598-022-09265-y)
Supplement: Supplementary file 1 — Supplementary Tables. [file 41598_2022_9265_MOESM1_ESM.docx]

| **Supplementary Table 1.** Polymorhpic sites analyzed to assign Caucasic and American mtDNA haplogroups | | | | | | | | | | | | | | |
| --- | --- | --- | --- | --- | --- | --- | --- | --- | --- | --- | --- | --- | --- | --- |
|  | **Polymorhpic sites** | | | | | | | | | | | | | |
| **Haplogroup** | Caucasic | | | | | |  | American | | | | | | |
|  | **t7028c** | **a10398g** | **a12308g** | **t4216c** | **t14766c** | **g4580a** |  | **a663g** | **t12705c** | **a13263g** | **c10400t** | **c5178a** | **c6371t** | **del8281:8289 a(CCCCCTCTA)g** |
| **A** | T | A | A | - | T | G |  | G | T | A | C | C | C | - |
| **B** | T | - | A | T | - | G |  | A | C | A | C | C | C | YES |
| **C** | T | A | A | T | T | G |  | A | T | G | T | C | C | NO |
| **D** | T | A | A | - | T | G |  | A | T | A | T | A | C | - |
| **X** | T | A | A | - | T | G |  | A | T | A | C | C | T | NO |
| **H** | C | A | A | T | C | G |  | A | C | A | C | C | C | NO |
| **V** | T | A | A | T | C | A |  | A | C | A | C | C | C | NO |
| **HV*** | T | A | A | T | C | G |  | A | C | A | C | C | C | NO |
| **U** | T | A | G | T | T | G |  | - | C | A | C | C | C | - |
| **K** | T | G | G | T | T | G |  | A | C | A | C | C | C | - |
| **T** | T | A | A | C | T | G |  | A | C | A | C | C | C | - |
| **J** | T | G | A | C | T | G |  | A | C | A | C | C | C | NO |
| Diagnostic SNP for each haplogroup | | |  |  |  |  |  |  |  |  |  |  |  |  |

| **Supplementary Table 2.** Primer sequences for polymerase chain reaction (PCR) and single base extension (SBE) reaction | | | | | | |
| --- | --- | --- | --- | --- | --- | --- |
| Polymorphic site | PCR primer |  | SBE primer | | | |
|  |  |  |  | | | |
| mt663a>g | F: 5´- ACCAAACCCCAAAGACACCC -3´ |  | 5´- CCCATAAACAAATAGGTTTGGTCCT -3´ | | | |
|  | R: 5´- GTGTGGCTAGGCTAAGCGTT -3´ |  |  |  |  |  |
|  |  |  |  | | | |
| mt12705t>c | F: 5´- ACTGAGCCACAACCCAAACA -3´ |  | 5'- tgttagcggtaactaagattagtatggtaattaggaa -3' | | | |
|  | R: 5´- CATCTGCTCGGGCGTATCAT -3´ |  |  |  |  |  |
|  |  |  |  | | | |
| mt13263a>g | F: 5´- GCTTAGGCGCTATCACCACT -3´ |  | 5'- aaagactgactgactgactgactcccccCGTAGCCTTCTCCACTTCAAGTCA -3' | | | |
|  | R: 5´- TCCTGCTAATGCTAGGCTGC -3´ |  |  |  |  |  |
|  |  |  |  | | | |
| mt10400c>t | F: 5´- GCCCTACAAACAACTAACCTGC -3´ |  | 5'- CTATGAGTGACTACAAAAAGGATTAGACTGAAC -3' | | | |
|  | R: 5´- TGTAAATGAGGGGCATTTGGT -3´ |  |  |  |  |  |
|  |  |  |  | | | |
| mt5178c>a | F: 5´- ACCAAACCCAGCTACGCAAA -3´ |  | 5'- gactgactgactgactgactCCTACTACTATCTCGCACCTGAAACAAG -3' | | | |
|  | R: 5´- AGGAGGGTGATGGTGGCTAT -3´ |  |  |  |  |  |
|  |  |  |  | | | |
| mt6371c>t | F: 5´- CGTTTCCCCGCATAAACAAC -3´ |  | 5´- ttgactgactgactgactgactgactgaTTCTCCTTACACCTAGCAGGTGTCTC -3´ | | | |
|  | R: 5´- GAAGGTGGTGTTGAGGTTGC -3´ |  |  |  |  |  |
|  |  |  |  | | | |
| del8281:8289  a(ccccctcta)g | F: 5´- CCACTTTCACCGCTACACGA -3´ |  |  | | | |
|  | R: 5´- AGGATTGTGGGGGCAATGAA -3´ |  |  | | | |
| PCR: polymerase chain reaction; F: forward; R: reverse; del: deletion; SBE: single base extension | | | | | | |
|  |  |  | |  |  |  |

| **Supplementary Table 3.** Analysis of the interaction between obesity and mtDNA haplogroup A in patients with knee OA | | | | | | | | | |
| --- | --- | --- | --- | --- | --- | --- | --- | --- | --- |
| **Categories** | **Healthy**  **(N=364)** | **OA**  **(N=353)** | **Total**  **(N=717)** | **B** | **OR** | **Interaction parameters** | | | |
| **No-obese + no-A (Ref)** | 170 (46.7) | 138 (39.1) | 308 (43.0) |  | 1 |  |  | **95% CI *** | |
| **Obese + no-A** | 46 (12.6) | 62 (17.6) | 108 (15.1) | 0.507 | 1.66 | **RERI** | 2.23 | 0.37 | 4.09 |
| **No-obese + Haplo A** | 124 (34.1) | 81 (22.9) | 205 (28.6) | -0.217 | 0.80 | **AP** | 0.60 | 0.34 | 0.87 |
| **Obese + Haplo A** | 24 (6.6) | 72 (20.4) | 96 (13.4) | 1.307 | 3.70 | **S** | 5.80 | 0.95 | 35.35 |
| Values are number of patients with percentage in parentheses; OR: odds ratio; B: regression coefficient; CI: confidence interval; (*) 95% CI calculated using Delta method by Hosmer & Lemeshow (1992); Ref: reference category; RERI: relative excess risk due to interaction; AP: attributable proportion due to interaction; S: synergy index | | | | | | | | | |

| **Supplementary Table 4.** Frequency distribution of American mtDNA haplogroups between osteoarthritic patients and healthy controls stratified by obesity status excluding subjects from Mexico city | | | | | | |
| --- | --- | --- | --- | --- | --- | --- |
| **Obesity status** | **mtDNA haplogroup** | **Healthy**  **(N=285)** | **OA**  **(N=223)** | **p-value*** | **OR** | **95% CI** |
| **Obese**  (BMI≥30 Kg/m^2^) | A | 21 (35.0) | 44 (57.1) | **0.010** | 2.48 | 1.23 – 4.97 |
|  | B | 11 (18.3) | 8 (10.4) | 0.182 | 0.51 | 0.19 – 1.38 |
|  | C | 12 (20.0) | 11 (14.3) | 0.375 | 0.67 | 0.27 – 1.64 |
|  | D | 7 (11.7) | 1 (1.3) | **0.010** | 0.10 | 0.01 – 0.83 |
|  | Others | 9 (15.0) | 13 (16.9) | 0.766 | 1.15 | 0.46 – 2.91 |
| Total |  | 60 | 77 |  |  |  |
| **No-obese**  (BMI<30 Kg/m^2^) | A | 95 (42.2) | 49 (33.6) | 0.094 | 0.69 | 0.45 – 1.07 |
|  | B | 47 (20.9) | 38 (26.0) | 0.250 | 1.33 | 0.82 – 2.17 |
|  | C | 36 (16.0) | 24 (16.4) | 0.911 | 1.03 | 0.59 – 1.82 |
|  | D | 18 (8.0) | 10 (6.8) | 0.682 | 0.85 | 0.38 – 1.89 |
|  | Others | 29 (12.9) | 25 (17.1) | 0.259 | 1.40 | 0.78 – 2.50 |
| Total |  | 225 | 146 |  |  |  |
| Values are number of patients with percentage in parentheses; BMI: body mass index; OR: odds ratio; CI: confidence interval; (*) statistical significance declared at p ≤0.01 (in bold) after Bonferroni correction | | | | | | |

| **Supplementary Table 5.** Multivariate logistic regression model to explore the effect of the interaction between obesity and mtDNA haplogroup A on the risk of knee OA excluding subjects from Mexico city | | | | |
| --- | --- | --- | --- | --- |
| **Variable** | **B** | **Adjusted OR** | **95% CI** | **p-value** |
| Age | 0.081 | 1.08 | 1.07 – 1.10 | **<0.001** |
| Gender (female) | 0.400 | 1.49 | 0.97 – 2.29 | 0.068 |
| Obesity | 0.433 | 1.54 | 0.84 – 2.83 | 0.161 |
| mtDNA haplogroup A | -0.143 | 0.87 | 0.52 – 1.43 | 0.577 |
| Obesity*mtDNA haplogroup A | 1.265 | 3.54 | 1.42 – 8.86 | **0.007** |
| BMI: body mass index; B: regression coefficient; OR: odds ratio; CI: confidence interval; statistical significance declared at p<0.05 (in bold) | | | | |
